# The relationship between tooth loss and mortality from all causes, cardiovascular diseases, and coronary heart disease in the general population: systematic review and dose–response meta-analysis of prospective cohort studies

**DOI:** 10.1042/BSR20181773

**Published:** 2019-01-11

**Authors:** Juxiang Peng, Jukun Song, Jing Han, Zhu Chen, Xinhai Yin, Jianguo Zhu, Jinlin Song

**Affiliations:** 1 Guiyang Hospital of Stomatology, Guiyang, China; 2 College of Stomatology, Chongqing Medical University, Chongqing, China; 3 Chongqing Key Laboratory for Oral Diseases and Biomedical Sciences, College of Stomatology, Chongqing Medical University, Chongqing, China; 4 Chongqing Municipal Key Laboratory of Oral Biomedical Engineering of Higher Education, College of Stomatology, Chongqing Medical University, Chongqing, China; 5 Department of Oral and Maxillofacial Surgery, Guizhou Provincial People’s Hospital, Guizhou 550002, China; 6 Department of Respiratory & Critical Care medicine, Guizhou Provincial People’s Hospital, Guizhou 550002, China; 7 Department of Urology, Guizhou Provincial People’s Hospital, Guizhou 550002, China

**Keywords:** CardioVascular Diseases, Coronary Heart Disease, Mortality, Systematic Review, Tooth Loss

## Abstract

**Background:** The association of tooth loss with mortality from all causes, cardiovascular diseases (CVD), and coronary heart disease (CHD) has been studied for many years; however, the results are inconsistent.

**Method:** PubMed, Embase, Web of Knowledge, and Cochrane Oral Health Group’s Trials Register databases were searched for papers published from 1966 to August 2018. We conducted dose–response meta-analysis to quantitatively evaluate the relation between tooth loss and risk of mortality from all causes, CVD, and CHD.

**Results**: In the present study, 18 prospective studies conducted until August 2018 were considered eligible for analysis. In the analysis of linear association, the summarized relative risk (RR) values for each 10-, 20-, and 32-tooth loss for all-cause mortality were 1.15 (1.11–1.19), 1.33 (1.23–1.29), and 1.57 (1.39–1.51), respectively. Subgroup and sensitivity analyses showed consistent results. A linear relationship was found among all-cause mortality, with *P*_nonlinearity_ = 0.306. The susceptibility to all-cause mortality increased by almost 1.48 times at very high tooth loss (28–32), and slight flattening of the curve was noted. However, the summarized RR values for increment for 10-, 20-, and 32-tooth loss were not or were marginally related to increased risk of mortality from CVD/CHD. Subgroup and sensitivity analyses revealed inconsistent results. Tooth loss showed linear association with CHD mortality but not with CVD mortality. The susceptibility to all-cause mortality increased by almost 1.48 and 1.70 times for CVD and CHD, respectively, at very high tooth loss (28–32). The curve exhibited slight flattening; however, no statistical significance was detected.

**Conclusion**: In the meta-analysis, our findings confirmed the positive relationship between tooth loss and susceptibility to all-cause mortality, but not for circulatory mortality. However, the finding that tooth loss might play a harmful role in the development of all-cause mortality remains inconclusive. Tooth loss may be a potential risk marker for all-cause mortality: however, their association must be further validated through large prospective studies.

## Introduction

Tooth loss plays an important role in human health [1] and significantly influences masticatory capacity, diet, nutrient intake, aesthetics, and food choice [2]. In adults, the number of tooth loss can be viewed as an index of lifetime accumulation of poor oral health mainly caused by dental caries and periodontal disease [3]. Evidence from observational studies shows that tooth loss may be associated with multiple adverse health effects, including epilepsy [4], cognitive impairment [5], ischemic heart disease [6], heart failure [6], stroke [7], peripheral vascular diseases [8], and cancer [9,10]. Epidemiological studies have been conducted to determine the association between tooth loss and susceptibility to mortality from all causes, cardiovascular diseases (CVD), and coronary heart disease (CHD). However, scholars reported conflicting results [6,11–27]. Previous studies that examined the association between tooth loss and mortality employed small sample sizes, leading to decreased statistical power. According to the report on Global Burden of Diseases, severe tooth loss affects 2% of the global population and is listed 36th among the most prevalent chronic diseases that affect life expectancy [28]. Given the high incidences of CVD and CHD, their economic costs to the society, and their potential effect on public health, we conducted a dose–response meta-analysis to explore the association of tooth loss with all-cause and cause-specific mortality. In this work, we aimed to clarify the strength of the relationship, the shape of the dose–response association curve, and the potential confounding factors of tooth loss and mortality. Elucidating this relationship may emphasize the importance of preventive methods for all-cause and cause-specific mortality.

## Methods

The study was reported according to the Preferred Reporting Items for Systematic Reviews and Meta-Analyses (PRISMA) Statement criteria [29].

### Search strategy

Literature searches were independently conducted by two of the authors (J.K.S. and J.X.P.) by using two strategies. A literature search was performed in PubMed, Embase, Web of Knowledge, and Cochrane Oral Health Group’s Trials Register databases for papers published from 1966 to August 2018 without restriction to regions, publication types, or languages. Eligible studies were identified through Boolean search formation using various combinations of Medical Subject Headings (MeSH) and non-MeSH terms: ‘tooth loss’ OR ‘tooth’ OR ‘teeth’ AND ‘mortality’ OR ‘survival’ OR ‘death’ and ‘fatal’ OR ‘lethal’ AND ‘cohort’ OR ‘prospective.’ After the electronic search, a manual search was also conducted through the reference lists from original research and review articles.

### Inclusion criteria and study selection

Prospective studies (cohort, case-cohort, and nested case–control studies) that investigated the association between tooth loss and mortality and employed a follow-up duration of 2 years or longer were included in the meta-analysis. Studies on mortality in specific populations, such as patients with end-stage kidney disease and diabetes, were excluded in the analysis. Retrospective case–control and cross-sectional studies were also excluded because of difficulty in drawing causal inferences. Adjusted relative risk (RR) was extracted in preference to non-adjusted RR; however, unadjusted RR and CI were calculated when the RR was not provided.

### Data extraction

Two authors (S.J.K. and C.Z.) used standardized tables to extract the data. We extracted the following information: first author, publication year, study design, country, age, duration of follow-up, sex, sample size, number of cases and participants, outcome ascertainment, assessment methods for tooth loss, and categories and multiple adjusted RR of tooth loss and corresponding 95% confidence interval (CI) for each category of exposure. Disagreements were resolved through discussion among the authors.

### Statistical analysis

We used RR as the effect measure for the association of tooth loss and mortality. Having a dentition of at least 20 teeth is associated with sufficient masticatory efficiency and is a stated health goal of the World Health Organization [30]. In addition, the maximum number of teeth was set to 32 because the third molars tend to induce inflammation. Thus, we calculated the summarized RR and 95% CI for each 10-, 20-, and 32-tooth loss by using a random-effect model. We also conducted a dose–response analysis by using the method proposed by Greenland and Longnecker [31]. The number of teeth lost was used as the median tooth loss. If the median tooth loss category was not available, the midpoint of the upper and lower boundaries was considered the dose of each category. Using the generalized least squares for trend estimation and restricted cubic spline, we explored a potential non-linear dose-response relationship between tooth loss and mortality, with four knots at 5%, 35%, 65%, and 95% of the distribution. We determined the best-fitting second-order fractional polynomial regression model as the one with the lowest deviance. A likelihood ratio test was used to assess the difference between the nonlinear and linear models to test for nonlinearity.

Subgroup and sensitivity analyses were conducted to investigate the potential sources of heterogeneity between studies. Heterogeneity was quantitatively assessed by *Q* test and *I*^2^. Subgroup analysis was stratified by sex, duration of follow-up, geographical location, number of cases, Newcastle–Ottawa Scale (NOS) quality, assessment of tooth loss, and adjustment for confounders (age, sex, smoking, alcohol consumption, physical activity, diabetes, hypertension, body mass index (BMI), socioeconomic status (SES), and marital status). Sensitivity analysis was performed by omitting one study in each turn and recalculating the summarized RR for the remaining studies.

According to the NOS scale, quality assessment was conducted for non-randomized studies. The full score was nine stars, and high-quality studies were defined as those with ≥6 stars.

Publication biases, such as small study effects, were evaluated by inspecting the funnel plot for asymmetry and using Begg’s and Egger’s tests (rank correlation and linear regression methods, respectively) [32,33]. If an obvious asymmetry was observed, then both fixed- and random-effect trim and fill models were employed for adjusted meta-analysis to examine whether the bias influenced the results [34]. Values with *P* < 0.05 were considered statistically significant. All statistical analyses were performed using Stata version 13.1 (StataCorp, College Station, TX, USA).

## Results

### Literature search and study characteristics

**Figure 1:**
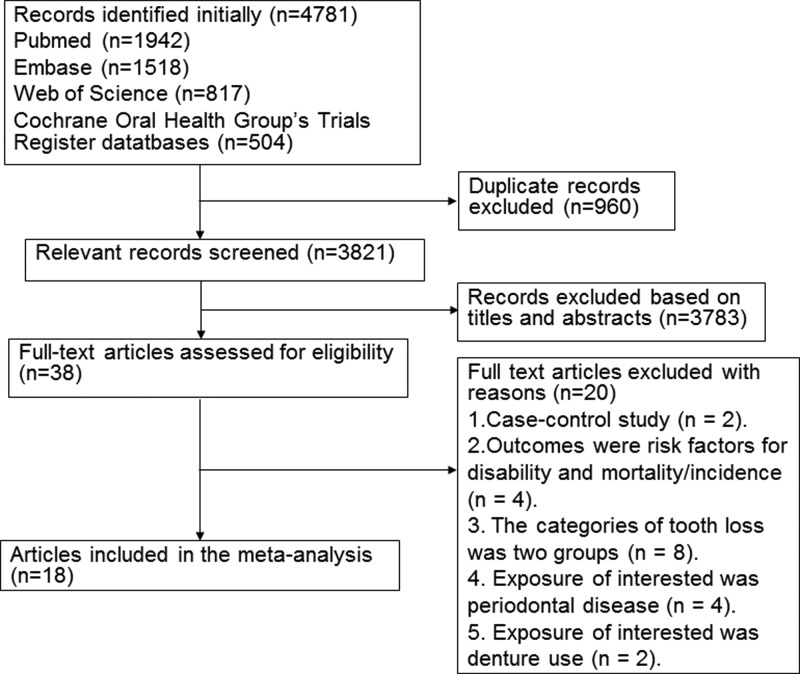
Flow chart of study selection

A diagram showing the details of study inclusion is shown in Figure 1. Following the development of our search strategy, 4781 records were screened. Of these, 960 records were excluded because they were duplicates, and 3783 records were excluded based on their titles and abstracts. Thus, 38 full-text articles were reviewed for further assessment. Two articles were excluded because they were case–control studies [35,36], and four articles were excluded because the outcome was disability/incidence of all-cause and CVD/CHD mortality [13,37–39]. Moreover, seven articles that employed two categories of exposure were excluded [40–46]. Four studies were also excluded because the exposure of interest was periodontal disease [47–50]. Furthermore, three studies were excluded because the exposure of interest was denture use [51,52] and the number of teeth was considered as a score variable [53]. Finally, we identified 18 prospective studies and included them in the analysis of tooth loss and mortality (Figure 1).

Eighteen prospective studies investigated the relationship between the number of teeth and all-cause mortality [6,11,12,14–17,19–25,27]. Seven studies investigated the effect on mortality from CHD [13,16,18,25,26] while five did so on CVD [13,16,18,25,26] (Table 1). These studies were published from 2003 to 2016. Among the 18 articles, nine studies were conducted in Europe [12,14,16,19–21,24–26], four in North America [13,18,22,23], three in Asia [11,15,17], one in Australia [6], and one across multiple countries [27]. We included a total of 19577 all-cause deaths, 1899 CVD-related deaths, and 1526 CHD-related deaths in the meta-analysis. The average follow-up duration ranged from 3.7 years to 57 years, the mean follow-up duration was 13.66 (standard deviation 12.12) years, and the median value was 12.00 (interquartile range, 6–15.8) years. Patients were followed up for over 5 years in the majority of the studies (84.2%).

**Table 1:** Characteristic of prospective cohort studies included in the meta-analysis.

Author, year	Country	Study name	Age, median (range), years	No. of cases	Cohort of size, gender	Outcome/end points ascertainment	Cause of death	Meassure of tooth loss	Duration of follow- up (years)	Adjustment for covariates
Hamalainen, 2003 [41]	Finland	The Evergreen project (Jyvaskyla, Finland)	NA (80–90)	150	226, male and female	Mortality data were received from the population register.	All-cause death	Clinical measured number of teeth	10	Adjusted for the general health variables.
Tuominen, 2003 [26]	Finland	Mini-Finland Health Survey	NA (30–69)	319	6527, male and female	Recorder linkage with the national mortality register.	CHD	Clinical measured number of teeth	12	Adjusted for age, other oral health indicators, level of education, hypertension, hypercholesterolemia, smoking, and diabetes.
Hung, 2004 [18]	USA	Health Professionals Follow-up Study (HPFS) and Nurses’ Health Study (NHS)	NA (40–75)	720	100381, male and female	The mortality records obtained from hospital records, autopsy report, or death certificate.	CHD	Self-reported number of teeth	12 (HPFS), 6 (NHS)	Adjusted for age (5-year categories), smoking (never, former, current, 1–14, 15–24, and ≥25 cigarettes per day), alcohol consumption (5 categories), body mass index (5 categories), physical activity (5 categories), family history of myocardial infarction, multivitamin supplement use, vitamin E use, history of hypertension, diabetes, and hypercholesterolemia in both cohorts and professions for men only, and for women only, menopausal status and hormone use.
Cabrera, 2005 [12]	Sweden	A prospective population study of women from Gothenburg	Middle-aged Swedish women	266	1462, female	Information on mortality was obtained from death certificates.	CVD, all-cause death	Clinical measured number of missing teeth	24	Adjusted for age and husband’s occupation, education, and income.
Tu, 2007 [25]	UK	Glasgow Alumni cohort	NA (≤30) (age at the entry of the study)	1635	12223, male and female	Recorder linkage with the national health service central register.	CVD and CHD, stroke, cancer, All-casue mortality	Clinical measured number of teeth	57	Adjusted for age at examination, sex, father’s socioeconomic position (derived from father’s occupation), smoking status, body mass index (BMI), computed as weight divided by height squared, and systolic blood pressure, all measured in early adulthood.
Dietrich, 2008 [13]	USA	VA Normative Aging and Dental Longitudinal Studies	NA (21–84)	109	1203, male	Triennial comprehensive medical examinations with the same criteria used in the Framingham Heart Study.	CHD	Clinical measured number of remaining teeth	24	Adjusted for age, body mass index, high-density lipoprotein cholesterol, total cholesterol, triglycerides, hypertension, mean systolic and diastolic blood pressure, diabetes mellitus, fasting glucose, smoking, alcohol intake, occupation and education, income, and marital status.
Padilha, 2008 [22]	USA	Baltimore Longitudinal Study of Aging (BLSA)	Survivors: 48.38 ± 14.52; decedents: 71.31 ± 11.10	198	500, male and female	Mortality ascertainment of inactive participants was done by telephone follow-up, correspondence from relatives, and annual searches of the National Death Index.	All-cause death	Clinical measured number of teeth	13.7 (164 months)	Adjusted for age, sex, self-rated health, glucose at 2 h, high-intensity physical activity, total physical activity, abdominal skinfold thickness, smoking, white blood cell count, myocardial infarction, cancer, clinical diabetes, angina, transitory ischemic attack, number of teeth, frequency of brushing teeth, difficulty of chewing, number of teeth with cervical caries, number of teeth with coronal caries, DMFT (sum of the number of decayed, missing and filled teeth), average Periodontal Index, and average Gingival Index.
Österberg, 2008 [21]	Sweden	Odontological cohorts (Goteborg)	70 (NA)	1003	1803, male and female	Mortality data were collected from the national Swedish health registers.	All-cause death	Clinical measured number of missing teeth	18	Adjusted for health factors, socio-economic and lifestyle factors.
Holmlund, 2011 [16]	Sweden		51.7 ± 13.8 (20–89)	629	7674, male and female	The date and cause of death were obtained from the Swedish Cause of Death Register using the unique personal number of all participating individuals.	CVD, CHD, Stroke, and all-cause death	Clinical measured number of remaining teeth	12	Adjusted for age, gender, and smoking.
Paganini-Hill, 2011 [23]	USA	The Leisure World Cohort Study	81 (52–105)	4753	5611, male and female	Search of government al and commercial death indexes and ascertainment of death certificates.	All-cause death	Self-reported number of natural teeth	9	Adjusted for age at entry, smoking, alcohol, caffeine, active activities, other activities, body mass index, high blood pressure, angina, heart attack, stroke, diabetes, rheumatoid arthritis, and cancer.
Hayasaka, 2013 [15]	Japan	The Ohsaki Cohort 2006 Study	NA (≥65)	2362	21730, male and female	Information on mortality was obtained from Ohsaki City government.	All-cause death	Self-reported number of teeth	4	Adjusted for age, sex, education level, smoking, alcohol drinking, body mass index, time spent walking daily, medical history, psychological distress, and energy and protein intake.
Schwahn, 2013 [24]	German	The Study of Health in Pomerania (SHIP)	63.6 (NA)	362	1803, male and female	Information was collected from population registries and local health authorities.	CVD, all-cause death	Clinical measured number of unreplaced teeth	9.9	Adjusted for age (restricted cubic splines), sex, education, marital status, partnership, smoking, risky alcohol consumption, physical activity, diagnosed diabetes mellitus, and obesity.
Ando, 2014 [11]	Japan	The Iwate-KENCO study	NA (40–79)	455	7779, male	Dates of death and relocation from the study area were annually or biannually confirmed by investigators who reviewed population -register sheets at each local government office.	CVD, cancer, all-cause death	Self-reported number of teeth	5.6	Adjusted for age, BMI, SBP, TC, HDLC, HbA1c, smoking status, alcohol drinking status, and education level.
Janket, 2014 [19]	Finland	Kuopio Oral Health and Heart (KOHH) study	60 (NA)	124	506, male and female	The mortality records obtained from the Finnish Death Registry.	CVD, all-cause death	Clinical measured number of teeth	15.8	Adjusted for age, sex, smoking (never, past, and current), hypertension, diabetes, total/HDL cholesterol ratio and education (in years), CRP ≥ 3 mg/l, fibrinogen > median (3.0 g/l).
Liljestrand, 2015 [20]	Finland	The National FINRISK 1997 Study	NA (25–74)	891	8446, male and female	Record linkage with the National Hospital Discharge register for hospitalizations and the disease-associated drug reimbursement records from the Social Insurance Institution of Finland.	All-cause death	Clinical measured number of missing teeth	13	Adjusted for age, sex, systolic blood pressure, total cholesterol (log), high-density lipoprotein (HDL) cholesterol (log), education (3 categories), smoking (yes/no), treated systolic blood pressure (medication within 0–7 days, yes/no), existing DM, and a geographic variable (east/west).
Hu, 2015 [17]	China (Taiwan)	A government-sponsored, annual physical examination program.	73.34 ± 6.8 (≥65) (NA)	3530	55651, male and female	Their mortality data were ascertained based on the national death files.	All-cause death	Clinical measured number of teeth	6	Adjusted for age, sex, marital status, education level, regular dental prophylaxis, smoking status, alcohol consumption, diabetes mellitus, hypertension, hyperlipidemia, and nonregular dental prophylaxis.
Vedin, 2015 [27]	39 countries from five continents.	STABILITY trial	64.4 ± 9.3 (NA)	1120	15456, male and female	All suspected endpoints were initially documented and reported by STABILITY study investigators and subsequently adjudicated according to pre-specified criteria by an independent clinical events committee, blinded with respect to the assigned treatment group.	CVD, all-cause death	Self-reported number of teeth	3.7	Adjusted for randomized treatment (darapladib or placebo), age, systolic BP, diastolic BP, body mass index, low-density lipoprotein cholesterol, high-density lipoprotein cholesterol, history of diabetes, prior MI, gender, smoking status and waist hip ratio, estimated glomerular filtration rate, family history of coronary heart disease, alcohol consumption, years of education, level of physical activity and country income level.
Joshy, 2016 [6]	Australia	The Sax Institute’s 45 and Up Study	NA (45–75)	1908	172630, male and female	Information on mortality was obtained from Centre for Health Record linkage.	ALL-cause death	Self-reported number of natural teeth left	3.9	Adjusted for age and sex, tobacco smoking, alcohol consumption, Australian born status, region of residence, education, health insurance, physical activity and body mass index, with missing values in covariates were coded as a separate categories (0.3%, 1.7%, 0%, 0.03%, 1.3%, 0%, 4%, and 7%, respectively).

Abbreviations: M, male; NA, not available; W, female.

All studies used clinically measured and self-reported numbers of teeth or missing teeth as measurement. Most studies used the number of remaining teeth, number of teeth, and number of missing teeth. Few studies employed the number of unreplaced teeth and number of natural teeth as exposure. The maximum number of teeth was set to 32. We transformed this information into the number of missing teeth. Most studies reported that death was either self-reported or identified from the national/regional cancer registries, which we assumed that the cancers were verified histologically. Many confounders (age, sex, social determinants such as SES and marital status, smoking, risky alcohol consumption, physical activity, metabolic factors such as diabetes and BMI, hypertension, oral health behavior, periodontitis, and caries) were considered in determining the relationship between the number of teeth and mortality. Most risk estimates were adjusted for age (*n* = 16) [6,11–13,15–20,22–27], smoking (*n* = 15) [6,11,13,15–20,22–27], diabetes mellitus (*n* = 9) [13,17–20,22–24,26,27], alcohol consumption (*n* = 9) [6,11,13,15,17,18,23,24,27], sex (*n* = 7) [6,15–17,19,20,22,24], and body mass index (*n* = 8) [6,11,13,15,18,23,25,27]. Some studies also controlled for physical activity (*n* = 6) [6,18,22–24,27], hypertension (*n* = 5) [13,17–19,26], and SES (*n* = 4) [12,13,21,25]. Few studies were adjusted for marital status (*n* = 3) [13,17,24], educational level (*n* = 2) [11,15], oral health behavior (*n* = 1) [22], and caries (*n* = 1) [22]. None of the studies were adjusted for environmental or genetic factors and periodontal disease.

### Quality of selected studies regarding the number of teeth and all-cause/circulatory mortality

We used NOS to evaluate the quality of the eligible studies (Table 2). The median NOS score was determined as 6.5 (range of 6–8). Analysis with high methodological quality was performed to determine the relationship between the number of teeth and mortality from all causes, CVD, and CHD.

**Table 2:** Quality assessment of included studies based on Newcastle–Ottawa Scale

Author	Year	Selection	Comparability	Exposure
Hamalainen, 2003 [41]	2003	3	1	2
Tuominen, 2003 [26]	2003	3	2	2
Hung, 2004 [18]	2004	3	2	3
Cabrera, 2005 [12]	2005	3	1	3
Tu, 2007 [25]	2007	2	1	3
Dietrich, 2008 [13]	2008	3	2	3
Padilha, 2008 [22]	2008	3	2	2
Österberg, 2008 [21]	2008	3	1	3
Holmlund, 2011 [16]	2011	3	1	3
Paganini-Hill, 2011 [23]	2011	3	2	3
Hayasaka, 2013 [15]	2013	3	1	2
Schwahn, 2013 [24]	2013	3	2	2
Janket, 2014 [19]	2014	3	1	3
Ando, 2014 [11]	2014	3	1	2
Liljestrand, 2015 [20]	2015	3	2	3
Hu, 2015 [17]	2015	3	2	2
Vedin, 2015 [27]	2015	3	2	2
Joshy, 2016 [6]	2016	3	2	2

### All-cause mortality

**Figure 2:**
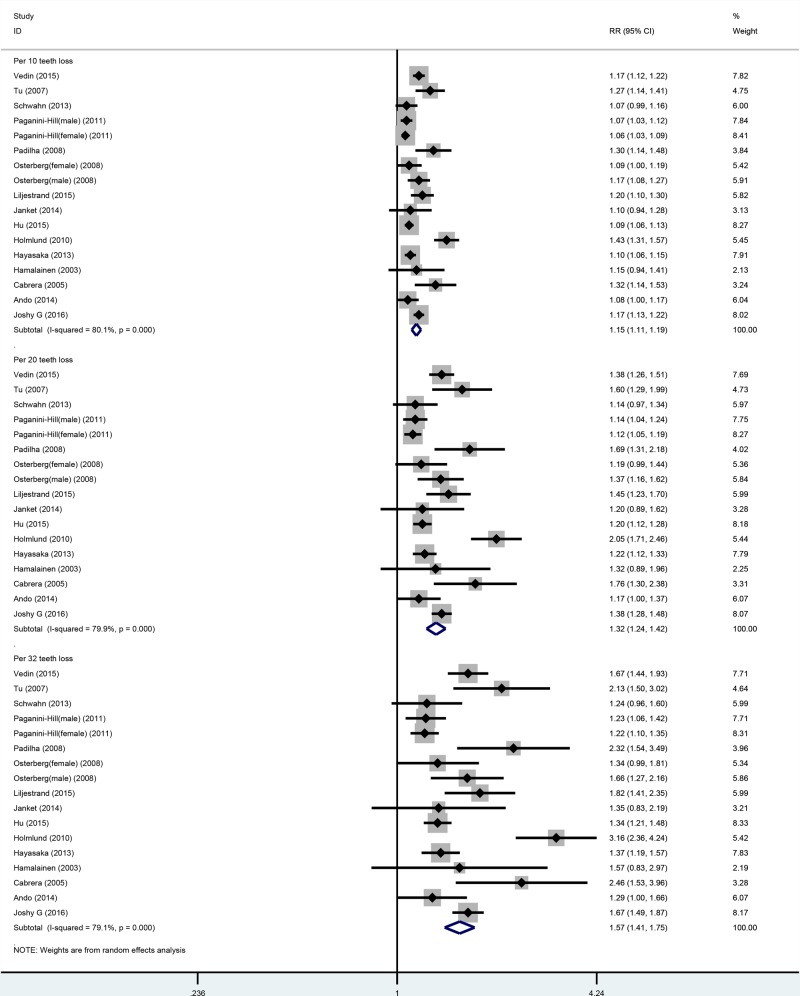
Linear dose–response analysis between tooth loss and all-causes mortality

Fifteen cohort studies were included in the dose–response analysis of the number of missing teeth and all-cause mortality. These studies involved a total of 19577 cases among 306807 participants [6,11,12,14–17,19–25,27]. In the linear or nonlinear dose–response analysis, the risk of all-cause mortality increased with increasing number of missing teeth. In the linear dose–response analysis, the summarized RR values for increment in 10-, 20-, and 32-tooth loss were 1.15 (1.11–1.19), 1.15 (1.11–1.19), 1.33 (1.24–1.42), and 1.57 (1.41–1.75), respectively (Table 3, Figure 2). In the sensitivity analysis, similar results were observed for all-cause mortality between 10-, 20-, and 32-tooth loss and all-cause mortality. The risk estimates ranged from 1.13 (95%CI: 1.10–1.16) with high heterogeneity (*I*^2^ = 70.6%, *P*_for heterogeneity_ = 0.000) (excluding the study by Holmlund et al. [16]), to 1.56 (95%CI: 1.11–1.20) with high heterogeneity (*I*^2^ = 80.8%, *P*_for heterogeneity_ = 0.000) (excluding the study by Hu et al.) for each 10-tooth loss. The risk estimates ranged from 1.28 (95%CI: 1.21–1.36) with high heterogeneity (*I*^2^ = 71.0%, *P*_for heterogeneity_ = 0.000) (excluding the study by Holmlund et al. [16]) to 1.34 (95%CI: 1.24–1.45) with significant heterogeneity (*I*^2^ = 80.6%, *P*_for heterogeneity_ = 0.000) (excluding the study by Hu et al. [17]) for every 20-tooth loss. The risk estimates ranged from 1.49 (95%CI: 1.36–1.64) with high heterogeneity (*I*^2^ = 69.3%, *P*_for heterogeneity_ = 0.000) (excluding the study by Holmlund et al. [16]) to 1.60 (95%CI: 1.42–1.81) with significant heterogeneity (*I*^2^ = 79.7%, *P*_for heterogeneity_ = 0.000) (excluding the study by Hu et al. [17]), for every 32-tooth loss.

**Table 3:** Meta-analysis of tooth loss and risk of all-causes mortality

All-causes mortality [RR (95%CI)]				
		10 teeth lost	20 teeth lost	32 teeth lost
**Total**		1.15 (1.11–1.19)	1.33 (1.24–1.42)	1.57 (1.41–1.75)
**Sex**				
Male		1.10 (1.04–1.16)	1.38 (1.27–1.51)	1.35 (1.14–1.60)
Female		1.12 (1.02–1.24)	1.27 (1.03–1.56)	1.48 (1.07–2.03)
Male + female		1.17 (1.12–1.23)	1.38 (1.27–1.51)	1.68 (1.46–1.93)
**Duration of follow-up**				
<10 years follow-up		1.11 (1.08–1.14)	1.23 (1.16–1.30)	1.39 (1.28–1.52)
≥10 years follow-up		1.26 (1.18–1.35)	1.59 (1.39–1.82)	2.11 (1.70–2.62)
**Geographic location**				
Europe		1.20 (1.12–1.28)	1.43 (1.25–1.64)	1.78 (1.43–2.21)
America		1.10 (1.03–1.17)	1.21 (1.06–1.39)	1.37 (1.10–1.70)
Asia		1.09 (1.07–1.12)	1.20 (1.14–1.27)	1.34 (1.24–1.45)
Australia		1.17 (1.13–1.22)	1.38 (1.28–1.48)	1.67 (1.49–1.87)
**Number of cases**				
Cases < 500		1.15 (1.07–1.24)	1.33 (1.14–1.55)	1.58 (1.24–2.02)
Cases 500–<1000		1.22 (1.09–1.35)	1.48 (1.20–1.84)	1.89 (1.35–2.65)
Cases ≥1000		1.12 (1.08–1.16)	1.26 (1.17–1.36)	1.44 (1.29–1.62)
**NOS quality**				
High (6–7 stars)		1.15 (1.11–1.19)	1.33 (1.24–1.42)	1.57 (1.41–1.75)
Low (0–6 stars)				
**Assessment of tooth loss**				
Self-reported number of teeth		1.11 (1.07–1.15)	1.23 (1.13–1.34)	1.40 (1.23–1.59)
Clinical measured number of teeth		1.19 (1.12–1.26)	1.42 (1.26–1.60)	1.75 (1.45–2.12)
**Adjustment for confounders**				
Age	Yes	1.15 (1.11–1.20)	1.33 (1.23–1.44)	1.59 (1.41–1.79)
	No	1.14 (1.07–1.20)	1.29 (1.15–1.45)	1.52 (1.26–1.83)
Gender	Yes	1.17 (1.11–1.23)	1.37 (1.23–1.53)	1.66 (1.40–1.97)
	No	1.13 (1.08–1.18)	1.28 (1.17–1.40)	1.49 (1.30–1.72)
Smoking	Yes	1.14 (1.10–1.18)	1.30 (1.20–1.40)	1.52 (1.35–1.72)
	No	1.19 (1.11–1.28)	1.42 (1.23–1.65)	1.77 (1.41–2.22)
Alcohol	Yes	1.09 (1.06–1.12)	1.20 (1.13–1.26)	1.34 (1.23–1.45)
	No	1.22 (1.15–1.30)	1.50 (1.32–1.70)	1.92 (1.57–2.35)
Physical activity	Yes	1.12 (1.07–1.18)	1.27 (1.14–1.41)	1.46 (1.25–1.72)
	No	1.17 (1.11–1.23)	1.37 (1.24–1.52)	1.66 (1.42–1.95)
Diabetes	Yes	1.11 (1.07–1.16)	1.25 (1.15–1.35)	1.43 (1.26–1.61)
	No	1.18 (1.13–1.23)	1.41 (1.26–1.57)	1.73 (1.45–2.06)
Hypertension	Yes	1.09 (1.05–1.12)	1.19 (1.11–1.28)	1.33 (1.19–1.48)
	No	1.18 (1.12–1.24)	1.39 (1.27–1.52)	1.70 (1.48–1.95)
BMI	Yes	1.12 (1.07–1.16)	1.25 (1.15–1.35)	1.43 (1.26–1.61)
	No	1.20 (1.12–1.29)	1.43 (1.22–1.67)	1.80 (1.44–2.24)
Socio-economics status	Yes	1.19 (1.10–1.29)	1.30 (1.21–1.40)	1.78 (1.39–2.27)
	No	1.14 (1.10–1.18)	1.43 (1.22–1.67)	1.53 (1.35–1.72)
Marital status	yes	1.09 (1.06–1.12)	1.19 (1.12–1.27)	1.33 (1.21–1.46)
	no	1.16 (1.12–1.21)	1.36 (1.25–1.47)	1.63 (1.42–1.85)

When stratifying the data into subgroups based on different exclusion criteria, similar results were obtained for all-cause mortality; however, the strength of the association differed among the studies (Table 3). Heterogeneity was very high in the overall and subgroup analyses. Asymmetry was observed in the funnel plot (Supplementary Figure S1), with *P*-values >0.05 for Begg’s test and *P* < 0.05 for Egger’s test, suggesting the existence of publication bias. We then used the trim-and-fill models for adjusted meta-analysis. Both fixed- and random-effect models demonstrated consistent results.

**Figure 3:**
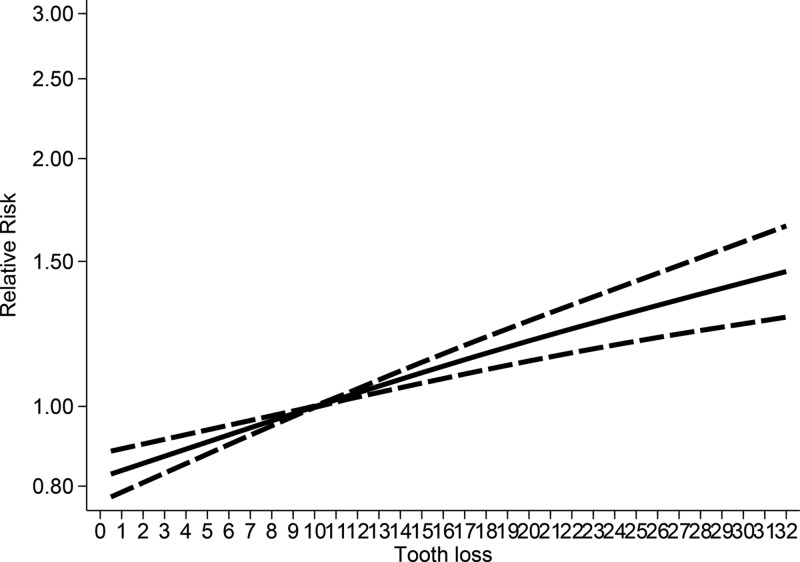
Nonlinear dose–response analysis between tooth loss and all-causes mortality

No evidence of nonlinear relationship was noted between tooth loss and all-cause mortality, with *P* for nonlinearity test = 0.306. An ‘X’ shape was observed between tooth loss and overall all-cause mortality risk, with a threshold level of 10 missing teeth. The association subsequently increased to around 10–32 missing teeth (Figure 3). The association was nonlinear for females (*P*_nonlinearity_ = 0.09, Supplementary Figure S2A) but linear for males (*P*_nonlinearity_ > 0.931, Supplementary Figure S2B).

### Cardiovascular disease

**Figure 4:**
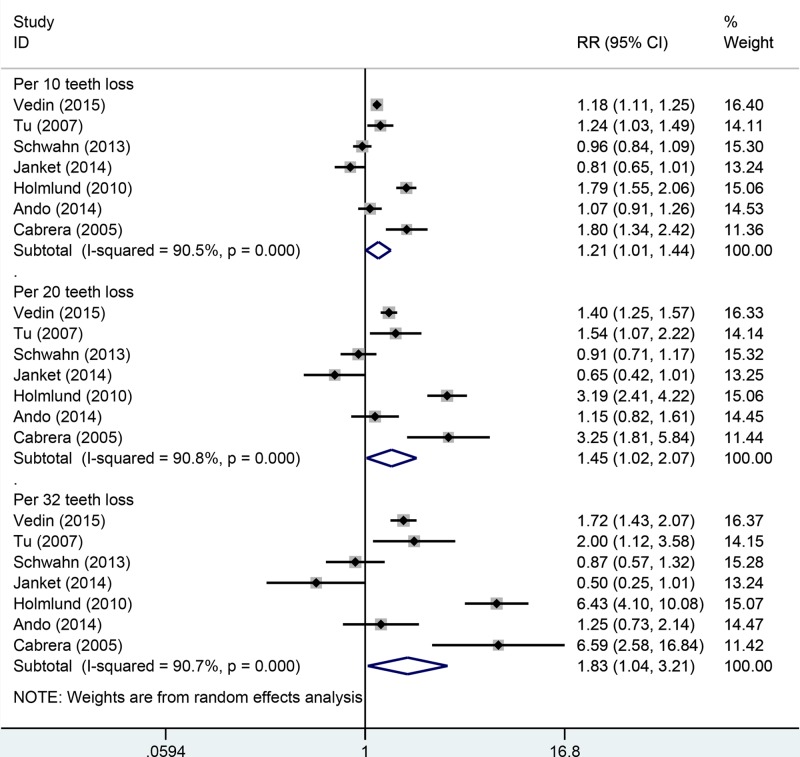
Linear dose–response analysis between tooth loss and CVD mortality

Seven cohort studies were included in the dose–response analysis of the number of missing teeth and CVD mortality; a total of 1899 cases were noted among 46130 participants [11,12,16,19,24,25,27]. In the linear dose–response analysis, increment in 10-, 20-, and 32-tooth losses was marginally associated with increased risk of CVD mortality. The risk of CVD mortality increased with increasing number of missing teeth. However, contradicting results were observed in the sensitivity and subgroup analyses (Table 4, Figure 4). Heterogeneity was very high in the overall and subgroup analyses. In the sensitivity analysis, inconsistent findings were observed between CVD mortality risk and increase per 10-, 20-, and 32-tooth loss. For 10-tooth loss, the RR ranged from 1.11 (95% CI: 0.97–1.29) with significant heterogeneity (*I*^2^ = 82.0%, *P*_for heterogeneity_ = 0.000) (excluding the study by Holmlund et al. [16]) to 1.28 (95% CI: 1.07–1.53) with significant heterogeneity (*I*^2^ = 90.2%, *P*_for heterogeneity_ = 0.000) (excluding the study by Janket et al. [19]). For 20-tooth loss, the RR ranged from 1.25 (95% CI: 0.93–1.68) with significant heterogeneity (*I*^2^ = 83.0%, *P*_for heterogeneity_ = 0.000) (excluding the study by Holmlund et al. [16]) to 1.64 (95% CI: 1.14–2.53) with significant heterogeneity (*I*^2^ = 90.5%, *P*_for heterogeneity_ = 0.000) (excluding the study by Janket et al. [19]). For 32-tooth loss, the RR ranged from 1.43 (95% CI: 0.90–2.29) with significant heterogeneity (*I*^2^ = 82.7%, *P*_for heterogeneity_ = 0.000) (excluding the study by Holmlund et al. [16]) to 2.21 (95% CI: 1.25–3.93) with significant heterogeneity (*I*^2^ = 90.4%, *P*_for heterogeneity_ = 0.000) (excluding the study by Janket et al. [19]).

**Table 4:** Meta-analysis of tooth loss and risk of CVD mortality

CVD mortality [RR (95%CI)]				
		10 teeth lost	20 teeth lost	32 teeth lost
**Total**		1.21 (1.01–1.47)	1.45 (1.02–2.07)	1.83 (1.04–3.21)
**Sex**				
Male		1.07 (0.91–1.36)	1.15 (0.82–1.61)	2.49 (1.18–5.27)
Female		1.80 (1.34–2.42)	3.25 (1.81–5.84)	1.67 (1.07–2.61)
Male+Female		1.16 (0.94–1.44)	1.34 (0.87–2.06)	1.44 (0.34–6.02)
**Duration of follow-up**				
<10 years follow-up		1.08 (0.94–1.23)	1.15 (0.86–1.54)	1.27 (0.80–2.00)
≥10 years follow-up		1.34 (0.93–1.93)	1.79 (0.87–3.70)	2.54 (0.79–8.14)
**Geographic location**				
Europe		1.25 (0.91–1.71)	1.55 (0.83–2.91)	2.03 (0.74–5.54)
Asia		1.07 (0.91–1.26)	1.15 (0.82–1.61)	1.25 (0.73–2.14)
**Number of cases**				
Cases < 500		1.08 (0.85–1.38)	1.17 (0.71–1.91)	1.29 (0.58–2.83)
Cases 500–<1000		1.79 (1.55–2.06)	3.19 (2.41–4.22)	6.43 (4.10–10.08)
Cases ≥1000		1.19 (1.12–1.25)	1.41 (1.26–1.58)	1.74 (1.46–2.08)
**NOS quality**				
High (6–7 stars)		1.21 (1.01–1.47)	1.45 (1.02–2.07)	1.83 (1.04–3.21)
Low (0–6 stars)				
**Assessment of tooth loss**				
Self-reported number of teeth		1.25 (0.91–1.71)	1.55 (0.83–2.91)	2.03 (0.74–5.54)
Clinical measured number of teeth		1.16 (1.08–1.25)	1.36 (1.18–1.56)	1.63 (1.28–2.06)
Adjustment for confounders				
Age	Yes	1.21 (1.01–1.47)	1.45 (1.02–2.07)	1.83 (1.04–3.21)
	No			
Gender	Yes	1.12 (0.70–1.80)	1.25 (0.48–3.24)	1.43 (0.31–6.60)
	No	1.24 (1.08–1.42)	1.54 (1.16–2.03)	1.99 (1.28–3.10)
Smoking	Yes	1.15 (0.96–1.37)	1.31 (0.91–1.89)	1.55 (0.87–2.77)
	No	1.80 (1.34–2.42)	3.25 (1.81–5.84)	6.59 (2.58–16.84)
Alcohol	Yes	1.08 (0.94–1.23)	1.15 (0.86–1.54)	1.27 (0.80–2.00)
	No	1.34 (0.93–1.93)	1.79 (0.87–3.70)	2.54 (0.79–8.14)
Physical activity	Yes	1.07 (0.88–1.93)	1.15 (0.75–1.75)	1.26 (0.65–2.44)
	No	1.28 (0.95–1.71)	1.63 (0.91–2.94)	2.19 (0.86–5.62)
Diabetes	Yes	0.99 (0.80–1.22)	0.98 (0.63–1.50)	1.41 (0.81–2.45)
	No	1.43 (1.09–1.87)	2.04 (1.19–3.50)	4.09 (0.76–22.01)
Hypertension	Yes	0.81 (0.65–1.01)	0.65 (0.42–1.01)	0.50 (0.25–1.01)
	No	1.28 (1.07–1.53)	1.61 (1.14–2.35)	2.21 (1.25–3.93)
BMI	Yes	1.11 (0.99–1.24)	1.22 (0.97–1.55)	1.91 (1.48–2.47)
	No	1.38 (0.81–2.34)	1.89 (0.65–5.45)	1.31 (0.16–10.49)
Socio-economics status	Yes	1.47 (1.02–2.11)	2.16 (1.04–4.47)	3.42 (1.07–10.95)
	No	1.13 (0.91–1.39)	1.27 (0.83–1.95)	1.47 (0.74–2.91)
Marital status	yes	0.96 (0.84–1.09)	1.58 (1.07–2.35)	4.15 (1.72–10.01)
	no	1.26 (1.03–1.53)	0.91 (0.71–1.17)	1.66 (0.84–3.26)

When stratifying the data into subgroups based on different exclusion criteria, inconsistent results for CVD mortality were obtained. In the subgroup analysis for sex, we found an increase in CVD mortality risk among the female population per 10-, 20-, and 32-tooth loss but not among the male population. When stratified by number of cases, tooth loss was significantly associated with increased risk of CVD mortality among cases >500. When we restricted the analysis to follow-up duration, geographical location, NOS quality, and adjustment for confounders, no significant association was observed. Both Begg’s test and Egger’s funnel plot asymmetry test (rank correlation test and regression method, respectively) in the meta-analysis indicated no significant publication bias (Begg’s test, *P* > 0.05; Egger’s test, *P* > 0.05). We also found little asymmetry in the funnel plot (Supplementary Figure S3).

**Figure 5:**
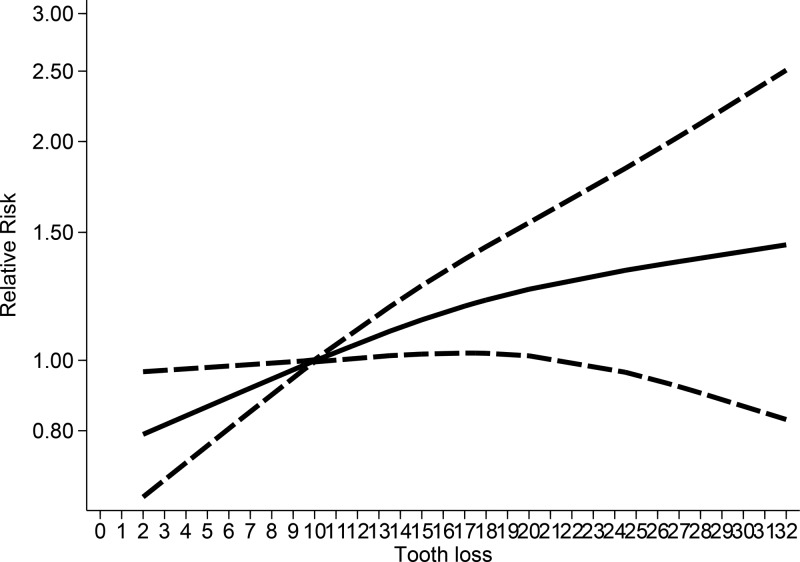
Nonlinear dose–response analysis between tooth loss and CVD mortality

No evidence of nonlinear association was observed between tooth loss and CVD mortality (*P* for nonlinearity test = 0.355). Tooth loss was not related to increased risk of CVD mortality below the level of 10 missing teeth, and the association marginally increased at around 10–19 missing teeth. Subsequent tooth loss increased the risk of all-cause mortality, with a slight flattening of the curve at around of 20–32 missing teeth; however, the statistical significance was not evident (Figure 5). Considering the few studies that were analyzed, we were not able to fit an interpretable nonlinear curve for males and females.

### Coronary heart disease

**Figure 6:**
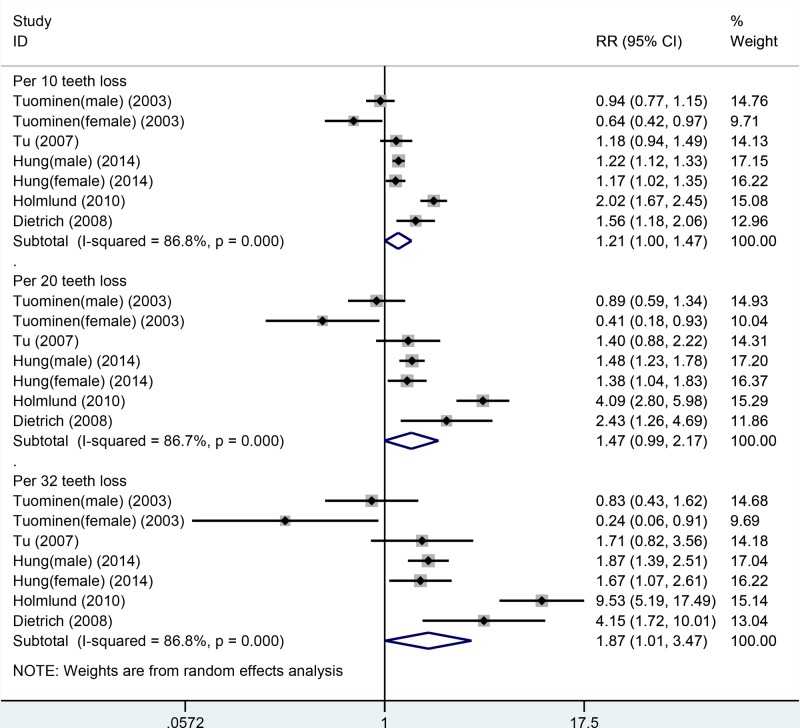
Linear dose–response analysis between tooth loss and CHD mortality

Five cohort studies were included in the dose–response analysis of the number of teeth and CHD mortality. A total of 1526 cases were included among 125716 participants [13,16,18,25,26]. In the dose–response analysis, an increment in each 32-tooth loss was marginally associated with increased risk of CHD mortality (RR: 1.87, 95% CI: 1.01–3.47) but not for 10- and 20-tooth loss. However, inconsistent results were observed in the sensitivity and subgroup analyses (Table 5, Figure 6). Heterogeneity was very high in the overall and subgroup analyses. In the sensitivity analysis, inconsistent findings were found between CHD mortality risk and each 10-, 20-, and 32-tooth loss. For 10-tooth loss, the RR ranged from 1.13 (95% CI: 0.97–1.30) with significant heterogeneity (*I*^2^ = 71.6%, *P*_for heterogeneity_ = 0.003) (excluding the study by Holmlund et al. [16]) to 1.30 (95% CI: 1.08–1.56) with significant heterogeneity (*I*^2^ = 85.9%, *P*_for heterogeneity_ = 0.000) (excluding the study by Tuominen et al. [26]). For 20-tooth loss, the RR ranged from 1.13 (95% CI: 0.97–1.30) with significant heterogeneity (*I*^2^ = 69.2%, *P*_for heterogeneity_ = 0.006) (excluding the study by Holmlund et al. [16]) to 1.30 (95% CI: 1.08–1.56) with significant heterogeneity (*I*^2^ = 85.7%, *P*_for heterogeneity_ = 0.000) (excluding the study by Tuominen et al. [26]). For 32-tooth loss, the RR ranged from 1.47 (95% CI: 0.93–2.34) with significant heterogeneity (*I*^2^ = 70.5%, *P*_for heterogeneity_ = 0.005) (excluding the study by Holmlund et al. [16]) to 2.33 (95% CI: 1.29–4.22) with significant heterogeneity (*I*^2^ = 85.9%, *P*_for heterogeneity_ = 0.000) (excluding the study by Tuominen et al. [26]).

**Table 5:** Meta-analysis of tooth loss and risk of CHD mortality.

CHD mortality [RR (95%CI)]				
		10 teeth lost	20 teeth lost	32 teeth lost
**Total**		1.21 (1.00–1.47)	1.47 (0.99–2.17)	1.87 (1.01–3.47)
**Sex**				
Male		1.33 (1.06–1.67)	1.71 (1.10-2.65)	2.49 (1.18–5.27)
Female		1.17 (1.02–1.35)	1.38 (1.04–1.83)	1.67 (1.07–2.61)
Male+female		1.12 (0.71–1.75)	1.25 (0.51–3.07)	1.44 (0.34–6.02)
**Duration of follow-up**				
<10 years follow-up		1.17 (1.02–1.35)	1.38 (1.04–1.83)	1.67 (1.07–2.61)
≥10 years follow–up		1.21 (0.95–1.55)	1.46 (0.89–2.41)	1.87 (0.85–4.11)
**Geographic location**				
Europe		1.12 (0.71–1.75)	1.25 (0.51–3.07)	1.44 (0.34–6.02)
America		1.24 (1.11–1.38)	1.50 (1.25–1.79)	2.00 (1.41–2.84)
**Number of cases**				
Cases <500		1.11 (0.94–1.32)	1.21 (0.86–1.71)	1.41 (0.81–2.45)
Cases 500–<1000		2.02 (1.67–2.45)	4.09 (2.80–5.98)	9.53 (5.19–17.49)
Cases ≥1000		1.18 (0.94–1.49)	1.40 (0.88–2.22)	1.71 (0.80–3.56)
**NOS quality**				
High (6–7 stars)		1.21 (1.00–1.47)	1.47 (0.99–2.17)	1.87 (1.01–3.47)
Low (0–6 stars)				
**Assessment of tooth loss**				
Self-reported number of teeth		1.21 (1.12–1.30)	1.45 (1.24–1.69)	1.81 (1.41–2.31)
Clinical measured number of teeth		1.20 (0.83–1.72)	1.43 (0.68–3.01)	1.80 (0.57–5.71)
**Adjustment for confounders**				
Age	Yes	1.21 (1.00–1.47)	1.47 (0.99–2.17)	1.87 (1.01–3.47)
	No			
Gender	Yes			
	No	1.21 (1.00–1.47)	1.47 (0.99–2.17)	1.87 (1.01–3.47)
Smoking	Yes	1.21 (1.00–1.47)	1.47 (0.99–2.17)	1.87 (1.01–3.47)
	No			
Alcohol	Yes	1.24 (1.11–1.38)	1.50 (1.25–1.79)	2.00 (1.41–2.84)
	No	1.12 (0.71–1.75)	1.25 (0.51–3.07)	1.44 (0.34–6.02)
Physical activity	Yes	1.21 (1.12–1.30)	1.45 (1.24–1.69)	1.81 (1.41–2.31)
	No	1.20 (0.83–1.72)	1.43 (0.68–1.71)	1.80 (0.57–5.71)
Diabetes	Yes	1.11 (0.94–1.32)	1.21 (0.86–1.71)	1.41 (0.81–2.45)
	No	1.55 (0.92–2.62)	2.41 (0.84–6.90)	4.09 (0.76–22.01)
Hypertension	Yes	1.11 (0.94–1.32)	1.21 (0.86–1.71)	1.41 (0.81–2.45)
	No	1.55 (0.92–2.62)	2.41 (0.84–6.90)	4.09 (0.76–22.01)
BMI	Yes	1.23 (1.13–1.33)	1.48 (1.28–1.70)	1.91 (1.48–2.47)
	No	1.09 (0.57–2.08)	1.18 (0.32–4.32)	1.31 (0.16–10.49)
Socio-economics status	Yes	1.34 (1.02–1.76)	1.75 (1.03–2.96)	2.57 (1.08–6.12)
	No	1.16 (0.91–1.48)	1.35 (0.82–2.22)	1.62 (0.73–3.60)
Marital status	yes	1.56 (1.18–2.06)	2.43 (1.26–4.69)	4.15 (1.72–10.01)
	no	1.17 (0.95–1.44)	1.35 (0.82–2.22)	1.66 (0.84–3.26)

When stratifying the data into subgroups based on different exclusion criteria, inconsistent results were obtained for CHD mortality. In the subgroup analyses for duration of follow-up, geographical location, and number of cases, we found an increase in CHD mortality risk in the subgroup analyses stratified by short duration of follow-up, America, and number of cases between 500 and 1000. When stratified by adjustment for age and sex, tooth loss was marginally associated with increased risk of CHD mortality for each 32-tooth loss, but not for each 10- and 20-tooth loss. When we restricted the analysis to adjustment for alcohol, physical activity, BMI, SES, and marital status, a positive association was observed. However, when stratified by adjustment for diabetes and hypertension, no significant association was observed. Both Begg’s test and Egger’s funnel plot asymmetry test (rank correlation test and regression method, respectively) in the meta-analysis indicated no significant publication bias (Begg’s test, *P* > 0.05; Egger’s test, *P* > 0.05). We also found little symmetry in the funnel plot (Supplementary Figure S4).

**Figure 7:**
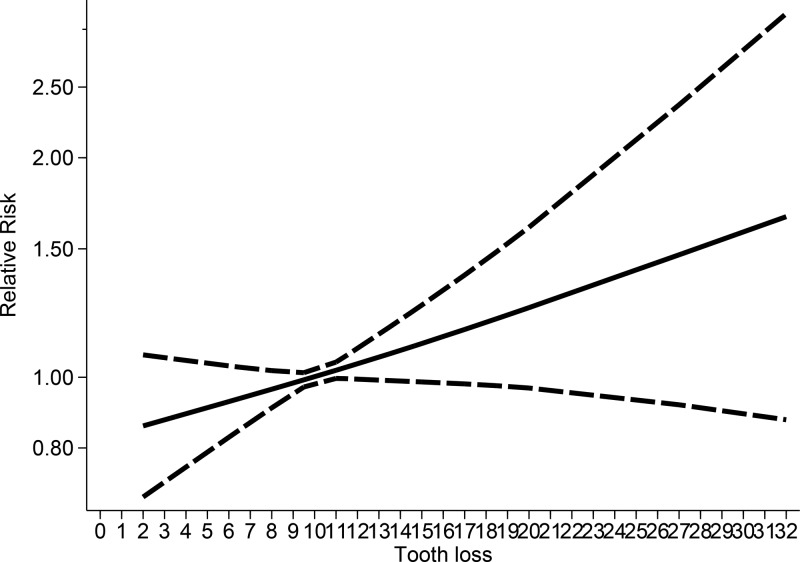
Nonlinear dose–response analysis between tooth loss and CHD mortality

No evidence of nonlinear association was observed between tooth loss and CHD mortality (*P* for nonlinearity test = 0.806). Tooth loss was not associated with increased risk of CHD mortality below the level of 10 missing teeth. Tooth loss was associated with marginally increased risk of CHD within 11–18 missing teeth and increased risk at around a tooth loss of 19–32; however, the statistical significance was not obvious (Figure 7). The association for males appeared to be nonlinear (*P*_nonlinearity_ = 0.819, Supplementary Figure S5). Considering the few studies evaluated, we were not able to fit an interpretable nonlinear curve for females.

## Discussion

To the best of our knowledge, the present study was the first to conduct nonlinear and linear dose–response meta-analysis for examining the association between tooth loss and risk of all-cause and circulatory mortality in the general population. In the linear dose–response analysis, we found 15%, 33%, and 57% increments in the relative risks of all-cause mortality per 10-, 20-, and 32-tooth loss; 21%, 45%, and 83% increments in the relative risk of CVD per 10-, 20-, and 32-tooth loss; and 21%, 47%, and 87% increments in the relative risk of CHD per 10-, 20-, and 32-tooth loss, respectively. Tooth loss was associated with increased risk of all-cause mortality. However, the associations between tooth loss and risk of CVD/CHD mortality were marginally statistically significant. The associations between tooth loss and all-cause mortality risk did not differ by different exclusion criteria and appeared to be linear. The positive associations observed in the meta-analysis confirmed the association between tooth loss and mortality from all causes but not for circulatory mortality. In the nonlinear analysis, the associations between tooth loss and all-cause mortality were not significant below 10 missing teeth however, there was an increase in the risk with 11–32 missing teeth, with a slight flattening of the curve at around 28–32 missing teeth. Based on the meta-analysis results, people who have lost more than 10 teeth should pay attention to increased risk of all-cause mortality; with increase in tooth loss, the risk of mortality also increased. However, the associations between tooth loss and CVD mortality were not significant within 0–10 missing teeth. The risk reached a slight plateau of the curve at around 11–19 missing teeth. The associations subsequently increased in risk with 20–32 missing teeth; however, the statistical significance was not obvious. The associations between tooth loss and CHD mortality were not significant below 10 missing teeth. The risk reached a slight plateau of the curve at around 11–18 missing teeth, and the risk increased at around a tooth loss of 19–32; however, the statistical significance was also not obvious.

As people age, they lose their teeth. Most of the missing teeth of an elderly subject would have been infected before tooth loss or extractions; hence, the number of missing teeth could be an indicator of poor oral health. Two major biological mechanisms could explain the link between tooth loss and mortality. First, infectious agents in oral health, such as *Streptococcus sanguinis* and *Actinobacillus actinomycetemcomitans*, exert possible direct effects contributing to the pathogenesis of atherosclerosis and thrombosis [54,55]. The associations between infectious agents found in the oral cavity and myocardial events are assumed to be reflected by the positive association observed between white blood cell count and serum albumin and myocardial events [56]. In addition, periodontal pathogens may affect CVD/CHD and lead to detrimental effects on endothelial function, atherosclerosis progression, and plaque stability [57]. Systemic inflammation could be a possible causative pathway based on observations of increased levels of inflammatory markers in patients with PD. This finding was also previously demonstrated in this cohort with higher levels of C-reactive protein among those with more tooth loss [58]. Moreover, periodontal therapy could result in reduced systemic inflammation and improved endothelial function; as such, the long-term effects of PD treatment on clinical outcomes are unknown [59]. Second, the negative impacts of tooth loss on daily activities such as phonation, altered diet, and socializing are well documented [60]. A severe consequence of tooth loss is edentulism, which is the complete absence of teeth within the oral cavity. This condition can deteriorate the individual’s quality of life. Moreover, altered diet choices may lead to malnutrition because of the insufficient mastication as a consequence of tooth loss. The associations between mortality and malnutrition are highlighted in literature [61]. Tooth loss may lead to an altered diet, which may include reduced intake of vegetables, dietary fiber, and whole-meal bread and could be directly proportional to increased mortality. Therefore, tooth loss could potentially increase the risk of all-cause and circulatory mortality.

This meta-analysis exhibited several strengths. Recall bias is not likely to explain our results and the possibility of selection bias is reduced because the meta-analysis was based on prospective studies. In addition, the first research highlight of this meta-analysis is its large sample size. The meta-analysis included a large number of cohort studies with more than 23000 cases among almost 1 million participants. The large number of total cases provided high statistical power to quantitatively evaluate the association between tooth loss and mortality risk. To increase comparability between studies, we conducted linear and nonlinear dose–response analyses. In addition, we expanded the meta-analysis and the association between different geographical regions and study designs, adjusted for covariates which were also explored. The result persisted in a number of subgroup and sensitivity analyses between tooth loss and all-cause mortality, suggesting that the findings were not likely to be due to the confounding and was robust to the influence of single studies. Second, publication bias is a potential concern in any meta-analysis because small studies with null results do not get published. However, in our meta-analysis, we found little evidence of publication bias analysis of tooth loss and risk of CVD/CHD mortality, but not for all-cause mortality. In addition, the further trim-and-fill method suggested no obvious changes in the results in both fixed- and random-effect model.

This meta-analysis presents several limitations that must be considered in interpreting the results.

First, the analysis of the length of the induction period allows for clarifying an exposure–outcome relationship and for falsifying the pathway assumed. Therefore, the observation period should cover a reasonable induction period. If a nutritional pathway was taken, an assumption, in which the effect of chewing disability on mortality for an induction period of at least 5 year, could be reasonable. The longer the supposed time sequence between exposure and the occurrence of the outcome, the more crucial to analyze the empirical induction period. Moreover, the accumulation of risk factors over a lengthy induction time complicates the exploration whether and to what extent is death a consequence of oral risk factors and to identify the underlying pathway. In the meta-analysis, the average follow-up ranged from 3.7 years to 57 years among included cohort studies. Patients were followed up for over 5 years in the majority of the studies (84.2%). Therefore, the observation period in the included cohort studies covered a reasonable induction period.

Second, all-cause mortality is a non-specific outcome related to ill health from multiple cases. Tooth loss may also reflect other non-disease-specific aspects, such as historical access to dental care and temporal and geographical trends in dental extractions. The determination of the specific causes of all-cause mortality and tooth loss was not possible. Despite the lack of this information, previous studies have shown that circulatory diseases, such as CVD and CHD, as well as cancer are the common causes of mortality among the elderly [62] and the main reasons for tooth loss are untreated dental caries and periodontal disease [63,64].

Third, tooth loss and mortality from all causes and CVD/CHD share common risk factors. Among these factors, age was strongly associated with tooth loss and mortality from all causes and CVD/CHD. Smoking and SES also had a considerable influence on the development of clinically detectable periodontal disease, which eventually over a period of time, leads to tooth loss. As smoking and SES are important risk factors for all-cause and circulatory mortality [65], the non-consideration or rough classification may led to overestimation of tooth-number-related effects on mortality. Similarly, not controlling diabetes, body mass index, or obesity as surrogates limits the validity of some findings because diabetes is an important risk factor for periodontal disease and strongly associated with increased mortality, thereby confounding the relationship between tooth loss and mortality [66]. Many, but not all, of the studies, had adjusted for potential confounding factors, although not all potential confounders were adjusted for in every study. Confounding bias may further explain the inconsistent patterns of the relationship between tooth loss and mortality.

Fourth, heterogeneity was common among the studies included in the meta-analysis; therefore exploring the potential sources of heterogeneity is essential. A significant heterogeneity was also detected in the study. Studies included in this meta-analysis were heterogeneous in terms of different populations investigated and diagnostic criteria for tooth loss, thereby contributing to the heterogeneity in the pooled analysis. Furthermore, unstable results were observed in subgroup and sensitivity analyses between tooth loss and CVD/CHD mortality, which indicated that more relevant articles are needed to further explore this association.

Finally, different measures and definitions of tooth loss were employed within the selected studies. Most studies used the number of remaining teeth, teeth loss, natural teeth, and unreplaced teeth as exposures. The number of remaining teeth was a non-specific marker of dental disease experience, whereas the number of unreplaced teeth may be considered to be a proxy for the current status of masticatory efficiency. The use of the number of teeth was more appropriate than the number of missing teeth as the exposure of interest. Therefore, the number of unreplaced teeth as exposure was preferred in the later studies. Most studies adopted self-reported tooth loss, whereas others used validated methods of examination by a professional dentist. The self-reported tooth loss was from study participants, which could have overestimated the average tooth loss. Self-reported tooth loss were validated measures of tooth loss [67] and were also useful instruments for data collection, particularly in large-scale epidemiological studies. Despite the use of self-reported information, sensitivity and subgroup analyses did not alter the results between tooth loss and all-cause mortality. However, in the subgroup and sensitivity analyses for CVD/CHD mortality, varying results were observed. No information on the duration of tooth loss, incident tooth loss during the follow-up period, denture use or implants was observed, which could potentially influence the results and constitute a source of misclassification. Overall, these limitations may affect our final conclusions.

In conclusion, our meta-analysis indicates that tooth loss, and in particular complete tooth loss (edentulism), could increase the risk of all-cause mortality. Tooth loss is a potential marker of all-cause mortality, but not for CVD/CHD mortality. However, we could not conclude in the present study that tooth loss may be a casual factor for all-cause mortality. In addition, because of the several common risk factors for oral and systemic diseases mentioned, careful interpretations of their relationship with mortality are needed. Additional large-scale and high-quality prospective studies are also required to evaluate the association between tooth loss and CVD/CHD mortality.

## Supporting information

**Supplementary Figure S1:** 

**Supplementary Figure S2:** 

**Supplementary Figure S3:** 

**Supplementary Figure S4:** 

**Supplementary Figure S5:** 

## Abbreviations

BMI: body mass index
CHD: coronary heart disease
CVD: cardiovascular diseases
NOS: Newcastle–Ottawa Scale
RR: relative risk
SES: socioeconomic status

## References

[1] Kamhi A.G., Gentry B., Mauer D. and Gholson B. (1990) Analogical learning and transfer in language-impaired children. J. Speech Hear. Disord. 55, 140–148 10.1044/jshd.5501.140 2299830

[2] Adegboye A.R., Twetman S., Christensen L.B. and Heitmann B.L. (2012) Intake of dairy calcium and tooth loss among adult Danish men and women. Nutrition 28, 779–784 10.1016/j.nut.2011.11.011 22459555

[3] Hull P.S., Worthington H.V., Clerehugh V., Tsirba R., Davies R.M. and Clarkson J.E. (1997) The reasons for tooth extractions in adults and their validation. J. Dent. 25, 233–237 10.1016/S0300-5712(96)00029-2 9175351

[4] John M.T. (2005) Patients with epilepsy may have an increased risk of tooth loss. J. Evid. Based Dent Pract. 5, 226–227 10.1016/j.jebdp.2005.09.010 17138383

[5] Gao W., Wang X., Wang X., Cai Y. and Luan Q. (2016) Association of cognitive function with tooth loss and mitochondrial variation in adult subjects: a community-based study in Beijing, China. Oral Dis. 22, 697–702 10.1111/odi.12529 27353124

[6] Joshy G., Arora M., Korda R.J., Chalmers J. and Banks E. (2016) Is poor oral health a risk marker for incident cardiovascular disease hospitalisation and all-cause mortality? Findings from 172 630 participants from the prospective 45 and Up Study BMJ Open 6, e012386 10.1136/bmjopen-2016-012386 27577588 PMC5013478

[7] Iwasaki M., Sato M., Yoshihara A., Ansai T. and Miyazaki H. (2016) Association between tooth loss and medical costs related to stroke in healthy older adults aged over 75 years in Japan. Geriatr. Gerontol. Int. 10.1111/ggi.12687 10.1111/ggi.12687 26799814

[8] Joshipura K. (2002) The relationship between oral conditions and ischemic stroke and peripheral vascular disease. J. Am. Dent. Assoc. 133, 23S–30S 10.14219/jada.archive.2002.0373 12085721

[9] Wang Y., Peng J., Li Y., Luo H., Huang G., Luo S. (2016) Association between tooth loss and risk of oesophageal cancer: a dose-response meta-analysis. Springerplus 5, 1020 10.1186/s40064-016-2711-6 27441139 PMC4938834

[10] Yin X.H., Wang Y.D., Luo H., Zhao K., Huang G.L., Luo S.Y. (2016) Association between tooth loss and gastric cancer: a meta-analysis of observational studies. PLoS One 11, e0149653 10.1371/journal.pone.0149653 26934048 PMC4774992

[11] Ando A., Tanno K., Ohsawa M., Onoda T., Sakata K., Tanaka F. (2014) Associations of number of teeth with risks for all-cause mortality and cause-specific mortality in middle-aged and elderly men in the northern part of Japan: the Iwate-KENCO study. Community Dent. Oral Epidemiol. 42, 358–365 10.1111/cdoe.12095 24476489

[12] Cabrera C., Hakeberg M., Ahlqwist M., Wedel H., Bjorkelund C., Bengtsson C. (2005) Can the relation between tooth loss and chronic disease be explained by socio-economic status? A 24-year follow-up from the population study of women in Gothenburg, Sweden Eur. J. Epidemiol. 20, 229–236 10.1007/s10654-004-5961-5 15921040

[13] Dietrich T., Jimenez M., Krall Kaye E.A., Vokonas P.S. and Garcia R.I. (2008) Age-dependent associations between chronic periodontitis/edentulism and risk of coronary heart disease. Circulation 117, 1668–1674 10.1161/CIRCULATIONAHA.107.711507 18362228 PMC2582144

[14] Hamalainen P., Meurman J.H., Keskinen M. and Heikkinen E. (2003) Relationship between dental health and 10-year mortality in a cohort of community-dwelling elderly people. Eur. J. Oral Sci. 111, 291–296 10.1034/j.1600-0722.2003.00055.x 12887393

[15] Hayasaka K., Tomata Y., Aida J., Watanabe T., Kakizaki M. and Tsuji I. (2013) Tooth loss and mortality in elderly Japanese adults: effect of oral care. J. Am. Geriatr. Soc. 61, 815–820 10.1111/jgs.12225 23590405

[16] Holmlund A., Holm G. and Lind L. (2010) Number of teeth as a predictor of cardiovascular mortality in a cohort of 7,674 subjects followed for 12 years. J. Periodontol. 81, 870–876 10.1902/jop.2010.090680 20350152

[17] Hu H.Y., Lee Y.L., Lin S.Y., Chou Y.C., Chung D., Huang N. (2015) Association between tooth loss, body mass index, and all-cause mortality among elderly patients in Taiwan. Medicine (Baltimore). 94, e1543 10.1097/MD.0000000000001543 26426618 PMC4616854

[18] Hung H.C., Joshipura K.J., Colditz G., Manson J.E., Rimm E.B., Speizer F.E. (2004) The association between tooth loss and coronary heart disease in men and women. J. Public Health Dent. 64, 209–215 10.1111/j.1752-7325.2004.tb02755.x 15562943

[19] Janket S.J., Baird A.E., Jones J.A., Jackson E.A., Surakka M., Tao W. (2014) Number of teeth, C-reactive protein, fibrinogen and cardiovascular mortality: a 15-year follow-up study in a Finnish cohort. J. Clin. Periodontol. 41, 131–140 10.1111/jcpe.12192 24354534 PMC3934352

[20] Liljestrand J.M., Havulinna A.S., Paju S., Mannisto S., Salomaa V. and Pussinen P.J. (2015) Missing teeth predict incident cardiovascular events, diabetes, and death. J. Dent. Res. 94, 1055–1062 10.1177/0022034515586352 25991651

[21] Osterberg T., Carlsson G.E., Sundh V. and Mellstrom D. (2008) Number of teeth – a predictor of mortality in 70-year-old subjects. Community Dent. Oral Epidemiol. 36, 258–268 10.1111/j.1600-0528.2007.00413.x 18474058

[22] Padilha D.M., Hilgert J.B., Hugo F.N., Bos A.J. and Ferrucci L. (2008) Number of teeth and mortality risk in the Baltimore Longitudinal Study of Aging. J. Gerontol. A Biol. Sci. Med. Sci. 63, 739–744 10.1093/gerona/63.7.739 18693229 PMC4984838

[23] Paganini-Hill A., White S.C. and Atchison K.A. (2011) Dental health behaviors, dentition, and mortality in the elderly: the leisure world cohort study. J. Aging Res. 2011, 156061 10.4061/2011/156061 21748004 PMC3124861

[24] Schwahn C., Polzer I., Haring R., Dorr M., Wallaschofski H., Kocher T. (2013) Missing, unreplaced teeth and risk of all-cause and cardiovascular mortality. Int. J. Cardiol. 167, 1430–1437 10.1016/j.ijcard.2012.04.061 22560949

[25] Tu Y.K., Galobardes B., Smith G.D., McCarron P., Jeffreys M. and Gilthorpe M.S. (2007) Associations between tooth loss and mortality patterns in the Glasgow Alumni Cohort. Heart 93, 1098–1103 10.1136/hrt.2006.097410 17164486 PMC1955024

[26] Tuominen R., Reunanen A., Paunio M., Paunio I. and Aromaa A. (2003) Oral health indicators poorly predict coronary heart disease deaths. J. Dent. Res. 82, 713–718 10.1177/154405910308200911 12939356

[27] Vedin O., Hagstrom E., Budaj A., Denchev S., Harrington R.A., Koenig W. (2016) Tooth loss is independently associated with poor outcomes in stable coronary heart disease. Eur. J. Prev. Cardiol. 23, 839–846 10.1177/2047487315621978 26672609

[28] Medina-Solis C.E., Perez-Nunez R., Maupome G. and Casanova-Rosado J.F. (2006) Edentulism among Mexican adults aged 35 years and older and associated factors. Am. J. Public Health 96, 1578–1581 10.2105/AJPH.2005.071209 16809586 PMC1551965

[29] Moher D., Liberati A., Tetzlaff J., Altman D.G. and Group P. (2009) Preferred reporting items for systematic reviews and meta-analyses: the PRISMA statement. BMJ 339, b2535 10.1136/bmj.b2535 19622551 PMC2714657

[30] Nguyen T.C., Witter D.J., Bronkhorst E.M., Pham L.H. and Creugers N.H. (2011) Dental functional status in a southern vietnamese adult population – a combined quantitative and qualitative classification system analysis. Int. J. Prosthodont. 24, 30–37 21209999

[31] Greenland S. and Longnecker M.P. (1992) Methods for trend estimation from summarized dose-response data, with applications to meta-analysis. Am. J. Epidemiol. 135, 1301–1309 10.1093/oxfordjournals.aje.a116237 1626547

[32] Begg C.B. and Mazumdar M. (1994) Operating characteristics of a rank correlation test for publication bias. Biometrics 50, 1088–1101 10.2307/2533446 7786990

[33] Egger M., Davey Smith G., Schneider M. and Minder C. (1997) Bias in meta-analysis detected by a simple, graphical test. BMJ 315, 629–634 10.1136/bmj.315.7109.629 9310563 PMC2127453

[34] Peters J.L., Sutton A.J., Jones D.R., Abrams K.R. and Rushton L. (2007) Performance of the trim and fill method in the presence of publication bias and between-study heterogeneity. Stat. Med. 26, 4544–4562 10.1002/sim.2889 17476644

[35] Lafon A., Tala S., Ahossi V., Perrin D., Giroud M. and Bejot Y. (2014) Association between periodontal disease and non-fatal ischemic stroke: a case-control study. Acta. Odontol. Scand. 72, 687–693 10.3109/00016357.2014.898089 24720864

[36] Johansson C.S., Ravald N., Pagonis C. and Richter A. (2014) Periodontitis in patients with coronary artery disease: an 8-year follow-up. J. Periodontol. 85, 417–425 10.1902/jop.2013.120730 23725030

[37] Holm-Pedersen P., Schultz-Larsen K., Christiansen N. and Avlund K. (2008) Tooth loss and subsequent disability and mortality in old age. J. Am. Geriatr. Soc. 56, 429–435 10.1111/j.1532-5415.2007.01602.x 18194226

[38] Oluwagbemigun K., Dietrich T., Pischon N., Bergmann M. and Boeing H. (2015) Association between number of teeth and chronic systemic diseases: a cohort study followed for 13 years. PLoS One 10, e0123879 10.1371/journal.pone.0123879 25945503 PMC4422697

[39] Joshipura K.J., Rimm E.B., Douglass C.W., Trichopoulos D., Ascherio A. and Willett W.C. (1996) Poor oral health and coronary heart disease. J. Dent. Res. 75, 1631–1636 10.1177/00220345960750090301 8952614

[40] Abnet C.C., Qiao Y.L., Dawsey S.M., Dong Z.W., Taylor P.R. and Mark S.D. (2005) Tooth loss is associated with increased risk of total death and death from upper gastrointestinal cancer, heart disease, and stroke in a Chinese population-based cohort. Int. J. Epidemiol. 34, 467–474 10.1093/ije/dyh375 15659476

[41] Hamalainen P., Meurman J.H., Kauppinen M. and Keskinen M. (2005) Oral infections as predictors of mortality. Gerodontology 22, 151–157 10.1111/j.1741-2358.2005.00064.x 16163906

[42] Osterberg T., Carlsson G.E., Sundh V. and Steen B. (2007) Number of teeth – a predictor of mortality in the elderly? A population study in three Nordic localities Acta Odontol. Scand. 65, 335–340 10.1080/00016350701739519 17965979

[43] Watt R.G., Tsakos G., de Oliveira C. and Hamer M. (2012) Tooth loss and cardiovascular disease mortality risk – results from the Scottish Health Survey. PLoS One 7, e30797 10.1371/journal.pone.0030797 22363491 PMC3282705

[44] Morita I., Nakagaki H., Kato K., Murakami T., Tsuboi S., Hayashizaki J. (2006) Relationship between survival rates and numbers of natural teeth in an elderly Japanese population. Gerodontology 23, 214–218 10.1111/j.1741-2358.2006.00134.x 17105502

[45] Brown D.W. (2009) Complete edentulism prior to the age of 65 years is associated with all-cause mortality. J. Public Health Dent. 69, 260–266 10.1111/j.1752-7325.2009.00132.x 19453862

[46] Aida J., Kondo K., Yamamoto T., Hirai H., Nakade M., Osaka K. (2011) Oral health and cancer, cardiovascular, and respiratory mortality of Japanese. J. Dent. Res. 90, 1129–1135 10.1177/0022034511414423 21730255

[47] Ajwani S., Mattila K.J., Narhi T.O., Tilvis R.S. and Ainamo A. (2003) Oral health status, C-reactive protein and mortality – a 10 year follow-up study. Gerodontology 20, 32–40 10.1111/j.1741-2358.2003.00032.x 12926749

[48] Ajwani S., Mattila K.J., Tilvis R.S. and Ainamo A. (2003) Periodontal disease and mortality in an aged population. Spec. Care Dentist. 23, 125–130 10.1111/j.1754-4505.2003.tb00297.x 14765890

[49] Appollonio I., Carabellese C., Frattola A. and Trabucchi M. (1997) Influence of dental status on dietary intake and survival in community-dwelling elderly subjects. Age Ageing 26, 445–456 10.1093/ageing/26.6.445 9466295

[50] Hujoel P.P., Drangsholt M., Spiekerman C. and Derouen T.A. (2001) Examining the link between coronary heart disease and the elimination of chronic dental infections. J. Am. Dent. Assoc. 132, 883–889 10.14219/jada.archive.2001.0300 11480641

[51] Yoshida M., Morikawa H., Yoshikawa M., Tsuga K. and Akagawa Y. (2005) Eight-year mortality associated with dental occlusion and denture use in community-dwelling elderly persons. Gerodontology 22, 234–237 10.1111/j.1741-2358.2005.00068.x 16329232

[52] Shimazaki Y., Soh I., Saito T., Yamashita Y., Koga T., Miyazaki H. (2001) Influence of dentition status on physical disability, mental impairment, and mortality in institutionalized elderly people. J. Dent. Res. 80, 340–345 10.1177/00220345010800010801 11269726

[53] Jansson L., Lavstedt S. and Frithiof L. (2002) Relationship between oral health and mortality rate. J. Clin. Periodontol. 29, 1029–1034 10.1034/j.1600-051X.2002.291108.x 12472996

[54] Herzberg M.C. and Meyer M.W. (1996) Effects of oral flora on platelets: possible consequences in cardiovascular disease. J. Periodontol. 67, 1138–1142 10.1902/jop.1996.67.10s.1138 8910832

[55] Mattila K.J., Valtonen V.V., Nieminen M.S. and Asikainen S. (1998) Role of infection as a risk factor for atherosclerosis, myocardial infarction, and stroke. Clin. Infect. Dis. 26, 719–734 10.1086/514570 9524851

[56] Grau A.J., Buggle F., Ziegler C., Schwarz W., Meuser J., Tasman A.J. (1997) Association between acute cerebrovascular ischemia and chronic and recurrent infection. Stroke 28, 1724–1729 10.1161/01.STR.28.9.1724 9303015

[57] Kebschull M., Demmer R.T. and Papapanou P.N. (2010) “Gum bug, leave my heart alone!” – epidemiologic and mechanistic evidence linking periodontal infections and atherosclerosis. J. Dent. Res. 89, 879–902 10.1177/0022034510375281 20639510 PMC3318075

[58] Paraskevas S., Huizinga J.D. and Loos B.G. (2008) A systematic review and meta-analyses on C-reactive protein in relation to periodontitis. J. Clin. Periodontol. 35, 277–290 10.1111/j.1600-051X.2007.01173.x 18294231

[59] Tonetti M.S., D’Aiuto F., Nibali L., Donald A., Storry C., Parkar M. (2007) Treatment of periodontitis and endothelial function. N. Engl. J. Med. 356, 911–920 10.1056/NEJMoa063186 17329698

[60] Gerritsen A.E., Allen P.F., Witter D.J., Bronkhorst E.M. and Creugers N.H. (2010) Tooth loss and oral health-related quality of life: a systematic review and meta-analysis. Health Qual. Life Outcomes 8, 126 10.1186/1477-7525-8-126 21050499 PMC2992503

[61] Lopes M.B., Silva L.F., Lopes G.B., Penalva M.A., Matos C.M., Robinson B.M. (2016) Additional contribution of the malnutrition-inflammation score to predict mortality and patient-reported outcomes as compared with its components in a cohort of African descent hemodialysis patients. J. Ren. Nutr. 27771304 10.1053/j.jrn.2016.08.006

[62] Murray C.J., Vos T., Lozano R., Naghavi M., Flaxman A.D., Michaud C. (2012) Disability-adjusted life years (DALYs) for 291 diseases and injuries in 21 regions, 1990-2010: a systematic analysis for the Global Burden of Disease Study 2010. Lancet 380, 2197–2223 10.1016/S0140-6736(12)61689-4 23245608

[63] Gaio E.J., Haas A.N., Carrard V.C., Oppermann R.V., Albandar J. and Susin C. (2012) Oral health status in elders from South Brazil: a population-based study. Gerodontology 29, 214–223 10.1111/j.1741-2358.2011.00617.x 22486627

[64] Chestnutt I.G., Binnie V.I. and Taylor M.M. (2000) Reasons for tooth extraction in Scotland. J. Dent. 28, 295–297 10.1016/S0300-5712(99)00069-X 10722904

[65] Kannel W.B. and Higgins M. (1990) Smoking and hypertension as predictors of cardiovascular risk in population studies. J. Hypertens. Suppl. 8, S3–S8 2286855

[66] Saremi A., Nelson R.G., Tulloch-Reid M., Hanson R.L., Sievers M.L., Taylor G.W. (2005) Periodontal disease and mortality in type 2 diabetes. Diabetes Care 28, 27–32 10.2337/diacare.28.1.27 15616229

[67] Coburn B.W., Sayles H.R., Payne J.B., Redman R.S., Markt J.C., Beatty M.W. (2015) Performance of self-reported measures for periodontitis in rheumatoid arthritis and osteoarthritis. J. Periodontol. 86, 16–26 10.1902/jop.2014.140339 25269524

