# Supplementary material for: The relationship between tooth loss and mortality from all causes, cardiovascular diseases, and coronary heart disease in the general population: systematic review and dose–response meta-analysis of prospective cohort studies
Source: Biosci Rep. 2019 Jan 11;39(1):BSR20181773. doi: 10.1042/BSR20181773 (PMC6328868; doi:10.1042/BSR20181773)
Supplement: Supplementary file 1 [file bsr20181773_Supp1.pdf]

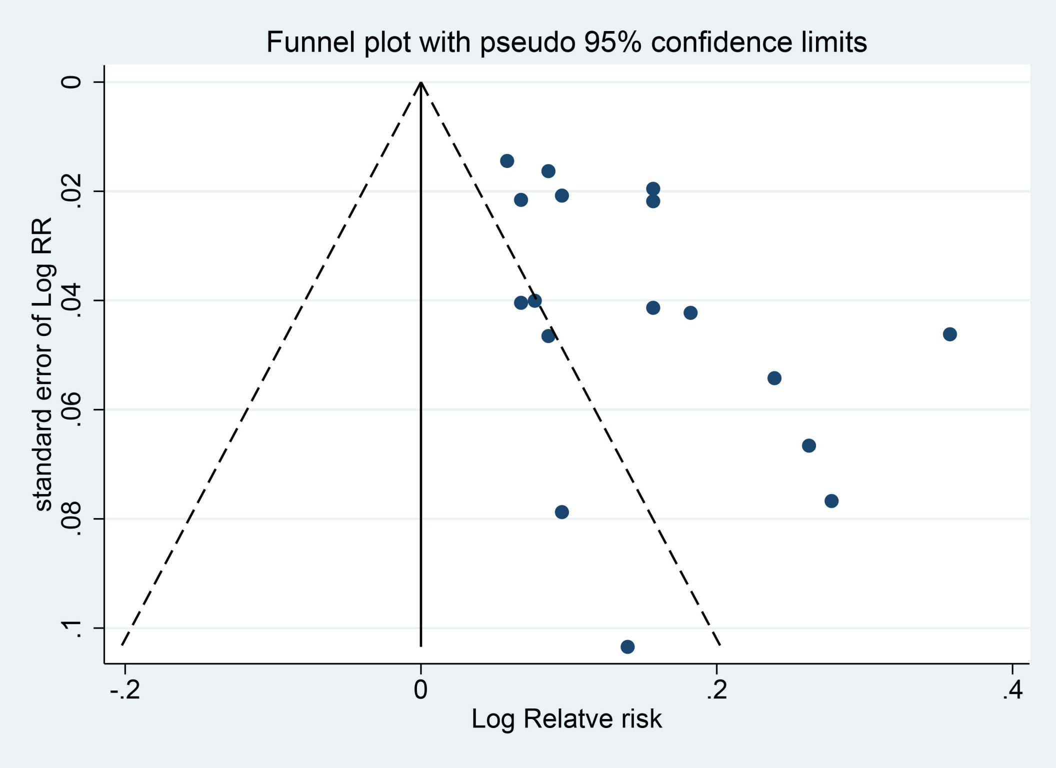

Funnel plot with pseudo 95% confidence limits
standard error of Log RR
0
.02
.04
.06
.08
.1
-.2
0
.2
.4
Log Relatve risk

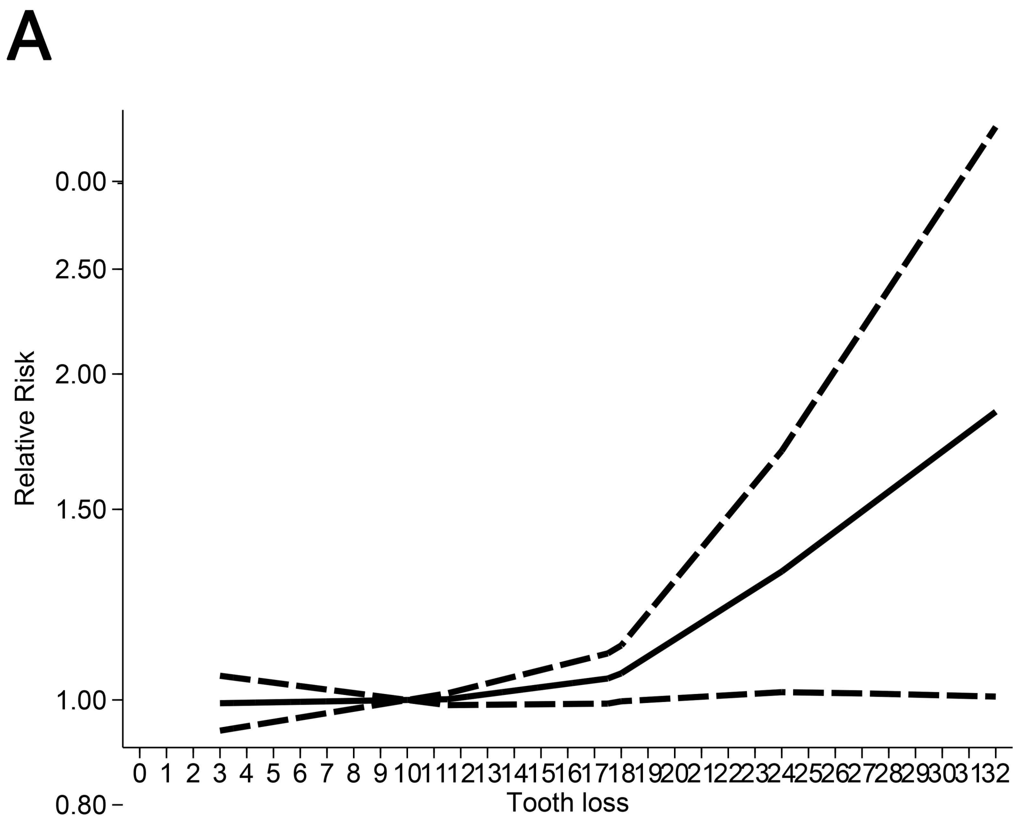

A
Relative Risk
0.00
2.50
2.00
1.50
1.00
0.80
0 1 2 3 4 5 6 7 8 9 10111213141516171819202122232425262728293031 32
Tooth loss

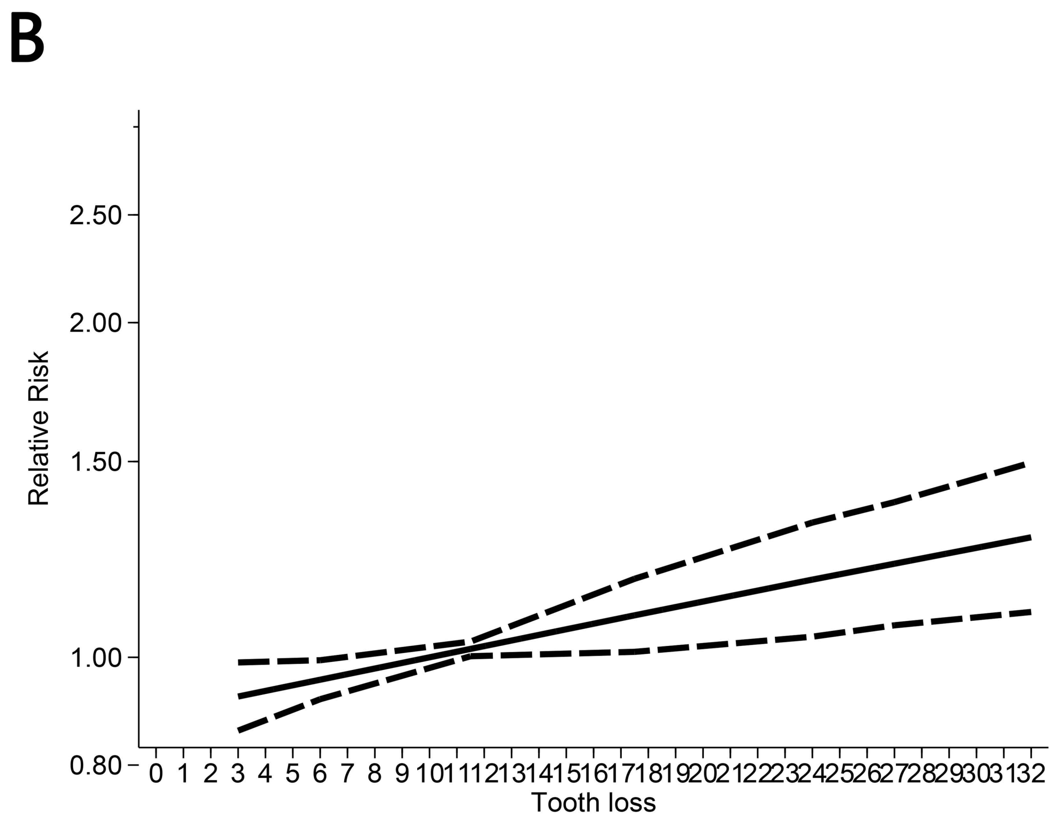

B
Relative Risk
2.50
2.00
1.50
1.00
0.80
0 1 2 3 4 5 6 7 8 9 10111213141516171819202122232425262728293031 32
Tooth loss

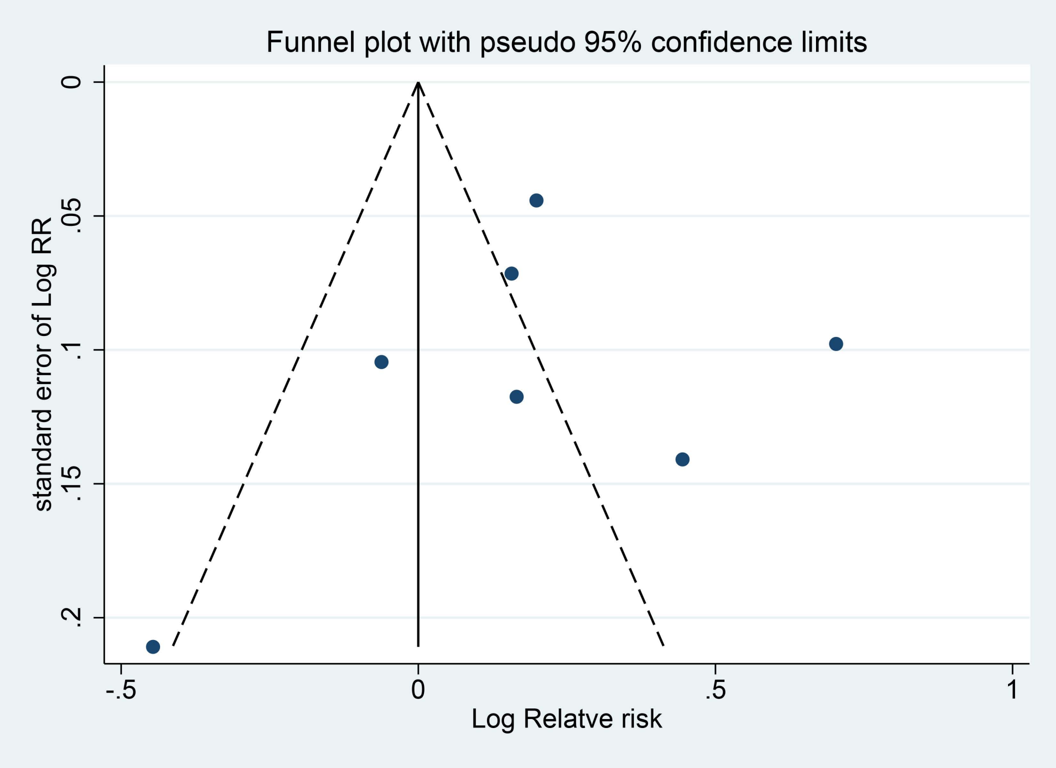

Funnel plot with pseudo 95% confidence limits
standard error of Log RR
0
.05
.1
.15
.2
-.5
0
.5
1
Log Relatve risk

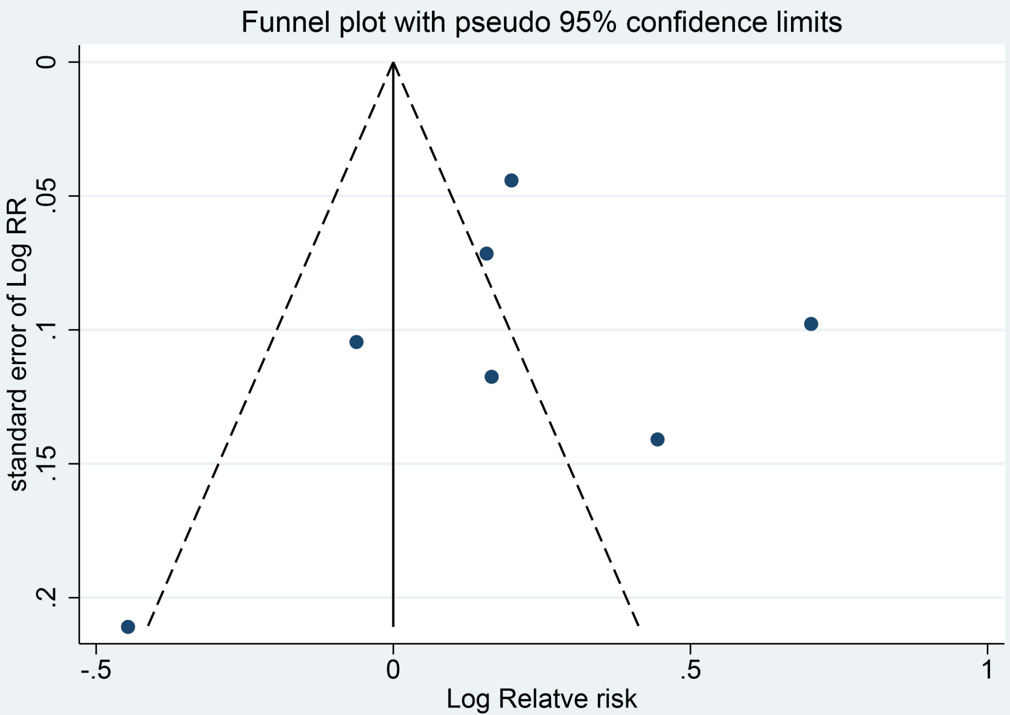

Funnel plot with pseudo 95% confidence limits
standard error of Log RR
0
.05
.1
.15
.2
-.5
0
.5
1
Log Relatve risk

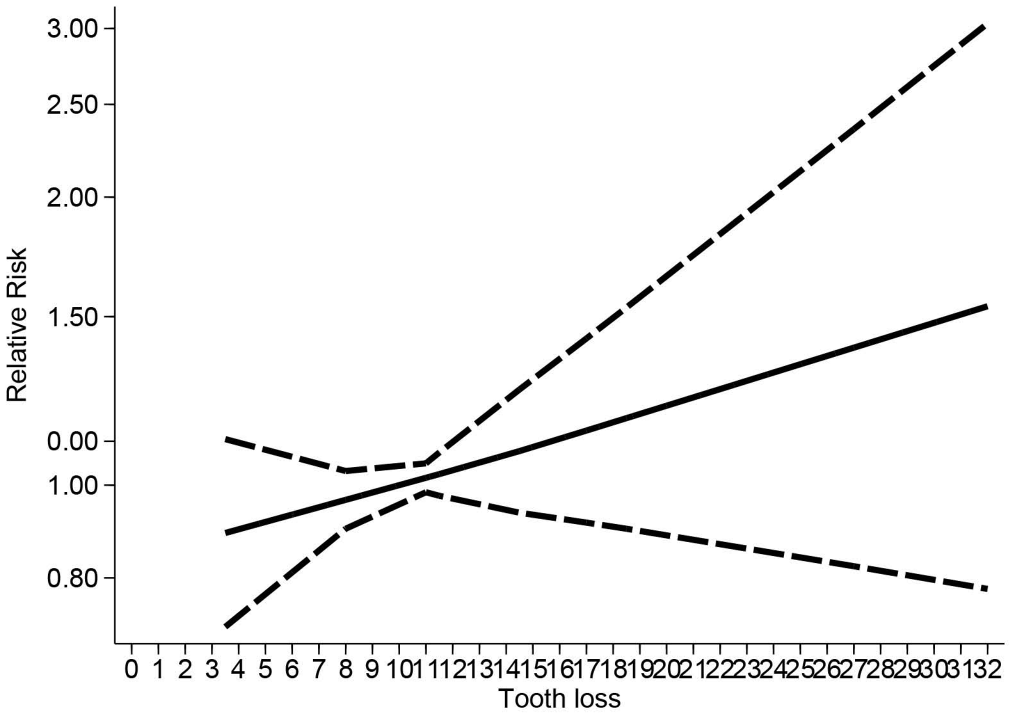

3.00
2.50
2.00
1.50
0.00
1.00
0.80
Relative Risk
0 1 2 3 4 5 6 7 8 9 10 11 12 13 14 15 16 17 18 19 20 21 22 23 24 25 26 27 28 29 30 31 32
Tooth loss
